# Feasibility and Pre–Post Changes Associated with a 12-Week Treadmill Walking Training Programme on Walking Performance, Physical Function, Fatigue, and Quality of Life in People with Multiple Sclerosis: A Single-Arm Pilot Study

**DOI:** 10.3390/healthcare14040552

**Published:** 2026-02-23

**Authors:** Gema Santamaría, Natalia Román Nieto, Raúl Cobreros Mielgo, Ana M. Celorrio San Miguel, Luis M. Cacharro, Juan F. Mielgo-Ayuso, Diego Fernández-Lázaro

**Affiliations:** 1 Department of Anatomy and Radiology, Faculty of Health Sciences, University of Valladolid, 42003 Soria, Spain; 2 Neurobiology Research Group, Faculty of Medicine, University of Valladolid, 47005 Valladolid, Spain; 3 Active Chronic Pain Management Unit, Sanidad Castilla y Leon (SACyL), 47011 Valladolid, Spain; 4 Physiotherapy Department, Institute of Biomedicine (IBIOMED), University of Leon, Campus de Vegazana, 24071 Leon, Spain; 5 Doctoral School, University of Leon, 24071 Leon, Spain; 6 Department of Ophthalmology of Salamanca University Assistance Complex (CAUSA), Salamanca University Hospital, 37007 Salamanca, Spain; 7 Department of Health Sciences, University of Burgos, 09001 Burgos, Spain; 8 Area of Histology, Faculty of Health Sciences, University of Valladolid, 42003 Soria, Spain

**Keywords:** multiple sclerosis, treadmill training, gait speed, timed up and go, short physical performance battery, fatigue, quality of life, pilot study

## Abstract

**Highlights:**

**What are the main findings?**

- A 12-week supervised treadmill walking training programme was feasible and safe for people with multiple sclerosis, with high adherence and no adverse events.
- The intervention was associated with improvements in walking performance, functional mobility, physical function, and fatigue, while overall quality of life showed domain-specific changes only.

**What are the implications of the main findings?**

- These findings suggest that supervised treadmill walking training can be integrated into routine neurorehabilitation settings and was associated with clinically meaningful pre–post changes in mobility and fatigue in people with multiple sclerosis.
- The domain-specific pre–post changes observed in vitality and general health suggest that treadmill-based exercise may preferentially be associated with changes in perceived energy levels and health status, rather than global quality of life, in people with multiple sclerosis.

**Abstract:**

**Background/Objectives**: Walking impairment and fatigue are common in multiple sclerosis (MS) and contribute to reduced physical function and quality of life (QoL). This study evaluated the feasibility, safety, and pre–post changes associated with a 12-week treadmill walking training (TWT) programme on walking performance, physical function, fatigue, and QoL in people with MS. **Methods**: Single-arm pilot study with pre–post assessments (T1–T2). Eleven adults with MS (Expanded Disability Status Scale [EDSS] ≤ 6) completed supervised TWT for 12 weeks (two 25 min sessions/week) at the Complejo Asistencial Universitario de Soria (Spain). Outcomes included SF-36, Timed Up and Go (TUG), 4 m gait speed, Short Physical Performance Battery (SPPB), and Modified Fatigue Impact Scale (MFIS). Within-participant changes were analysed using paired *t*-tests or Wilcoxon signed-rank tests as appropriate; effect sizes were reported as appropriate for the statistical test. **Results**: SF-36 total score did not change significantly (*p* = 0.160), while general health (*p* = 0.039) and vitality (*p* = 0.043) improved. Walking performance improved (TUG, *p* = 0.007; 4 m gait speed, *p* < 0.001), and physical function increased (SPPB, *p* = 0.003). Fatigue impact decreased (MFIS total, *p* = 0.015; physical, *p* = 0.007; psychosocial, *p* = 0.026), whereas the cognitive subscale did not change significantly (*p* = 0.094). Adherence was 91.7%, and no adverse events were reported. **Conclusions**: In this pilot sample, a 12-week TWT programme was feasible and safe and was associated with improvements in walking performance, physical function, and fatigue, with QoL changes limited to specific SF-36 domains. These findings support proceeding to a randomised controlled trial to establish efficacy. These findings should be interpreted as preliminary and exploratory, given the single-arm pre–post study design.

## 1. Introduction

Multiple sclerosis (MS) is a chronic inflammatory demyelinating disease of the central nervous system that presents with a heterogeneous combination of motor, sensory, cognitive and psychological symptoms, leading to substantial functional limitations and reduced quality of life (QoL) [1,2]. Motor impairments such as muscle weakness, spasticity and impaired coordination frequently compromise gait, while fatigue and cognitive symptoms further limit daily functioning and participation [2,3,4,5]. Walking impairment is highly prevalent in MS, affecting more than 75% of people with the disease, and it tends to worsen over time, restricting autonomy, social and occupational participation, and ultimately QoL [6,7]. Common gait alterations include reduced gait speed and cadence, shorter stride length and increased step-to-step variability [8,9], features that are clinically meaningful because gait speed is strongly related to functional independence and mobility capacity [10].

Fatigue is one of the most frequent and disabling symptoms in MS and is associated with poorer mental and general health, reduced employment, mobility limitations and restricted social participation [11,12]. Importantly, fatigue may contribute to a cascade of reduced physical activity and deconditioning, increasing fall risk, impairing balance and potentially accelerating the development of frailty-related vulnerability [13,14,15]. Consistent with this, sedentary behaviour is common in MS and is influenced by multiple factors, including fear of symptom worsening and fear of falling [16,17]. Reduced activity can initiate a self-reinforcing cycle of deconditioning, declining physical function, increasing frailty and decreased QoL [4,18,19,20,21]. Because frailty reflects reduced physiological reserve and increased vulnerability to stressors, its assessment and modification may provide a clinically relevant framework to capture multidimensional disability risk beyond isolated measures of gait or fatigue [7,14].

Exercise-based rehabilitation is a cornerstone of non-pharmacological care in MS and is generally considered safe and beneficial when appropriately prescribed [4,22]. Exercise training can reduce fatigue, improve mood, and support mechanisms related to neuroprotection and neuroplasticity, potentially contributing to maintenance or recovery of motor and cognitive function [4,22]. However, benefits may vary across disease phenotypes and disability levels, and long-term adherence remains challenging, especially among those with progressive disability or higher fear of falling [16,19,20,21,23]. Therefore, interventions that are feasible, scalable and task-specific for walking function are particularly relevant for clinical translation.

Treadmill walking training (TWT) enables structured and progressive repetition of the gait cycle in a controlled environment, with the ability to adjust intensity through speed and other parameters, making it a widely used task-specific approach for improving walking ability [24,25]. TWT may also enhance stability and confidence, particularly when handrails are available, potentially reducing concern for postural control during training [25]. In MS, TWT interventions—alone or combined with technologies such as virtual reality—have shown potential to improve gait performance and, in some cases, fatigue and cognitive outcomes [8,9,26,27,28,29]. However, existing evidence remains heterogeneous and, in some cases, limited by small samples, variable training doses, and focus on a narrow set of outcomes [8,9,22,26,27]. Additionally, some improvements may diminish when training is discontinued, underscoring the importance of designing interventions with feasible implementation and sustained engagement [30].

Notably, prior studies have primarily emphasised gait speed, fatigue, or QoL outcomes, while broader clinical constructs such as frailty and multidimensional vulnerability have been less frequently incorporated into treadmill-based interventions in MS [7,22]. Furthermore, it remains unclear whether short-term improvements in objective functional measures translate into meaningful changes in QoL, which is strongly influenced by psychosocial and disease-related factors beyond physical performance alone [31,32]. Addressing these gaps may help refine rehabilitation targets and identify clinically meaningful outcomes that better reflect the complexity of disability trajectories in MS. Therefore, the primary objective of this pilot study was to evaluate the observed pre–post changes associated with a 12-week TWT programme on quality of life, gait speed (including its role as a frailty-related indicator), physical function, and fatigue in people with MS, as well as to assess the feasibility and safety of the protocol in a hospital setting. Frailty was not assessed as a multidimensional clinical syndrome; rather, gait speed was examined specifically as a frailty-related slowness indicator, consistent with commonly used operational definitions of mobility-related vulnerability.

## 2. Materials and Methods

### 2.1. Ethical Considerations

The Clinical Ethics and Research Committee (CEIC) of the University of León (Spain) approved the study protocol (ETICA-ULE-010-2020) (Appendix A). The study was conducted in accordance with the Declaration of Helsinki (2008) and its Fortaleza update (2013) [33]. All participants provided written informed consent prior to participation and received a copy of the consent form. The trial was registered in a World Health Organization (WHO)-approved public registry: the Australian New Zealand Clinical Trial Registry (ANZCTR; ACTRN12622000264785).

### 2.2. Study Design

A single-arm pilot study with a pre–post design was conducted to evaluate the feasibility, safety, and preliminary effects of a 12-week TWT programme on QoL, walking performance (assessed by Timed Up and Go [TUG] and 4 m gait speed), physical function, and fatigue in people with MS. This design is appropriate for pilot studies where primary aims include process evaluation (adherence, retention, and safety) and estimation of variability and effect sizes to inform the design of a subsequent randomised controlled trial [34].

The study was conducted as part of an institutional implementation and validation initiative to inform the potential integration of the TWT programme into the regional health service portfolio (SACyL, Castilla y León, Spain). All procedures (recruitment, assessments, training) took place at the Complejo Asistencial Universitario de Soria.

**Figure 1:**
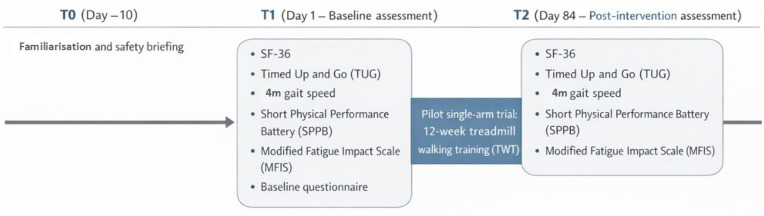
Study timeline and assessment points of the single-arm pilot trial. T0: familiarisation (~Day–10). T1: baseline (Day 1). T2: post-intervention (Day 84). Abbreviations: MFIS, Modified Fatigue Impact Scale; SPPB, Short Physical Performance Battery; TUG, Timed Up and Go.

Timeline and assessments: T0 (~Day–10), familiarisation and safety briefing; T1 (Day 1), baseline assessment (all outcomes plus sociodemographic/clinical questionnaire); and T2 (Day 84), post-intervention assessment (all outcomes). To minimise circadian influences, T1 and T2 assessments were conducted at 10:00 a.m. under identical conditions. At baseline, participants completed a structured sociodemographic and clinical questionnaire capturing age, sex, years since diagnosis, disability-related information, Expanded Disability Status Scale (EDSS), MS phenotype, medication use, medical history, and sleep quality (Appendix B). The timeline is summarised in Figure 1.

Reporting followed Consolidated Standards of Reporting Trials (CONSORT) principles where applicable (Appendix C) [35]. Given the pilot nature, the sample size supported feasibility assessment and estimation of variability/effect sizes rather than hypothesis testing. G*Power (version 3.1.9.6) was used for planning [36]; analyses were exploratory, focusing on within-participant changes and effect size estimation [34]. Feasibility outcomes included adherence (≥80% session attendance), retention (T2 completion), and data completeness.

All T1 and T2 assessments were performed by the same unblinded assessor to minimise inter-rater variability. Outcomes were analysed as within-participant changes (T1 to T2).

Accordingly, the primary aims of this pilot study were to assess feasibility and safety and to estimate variability and effect sizes to inform the design of future randomised controlled trials, rather than to establish intervention efficacy.

### 2.3. Participants

Spanish adults diagnosed with MS were recruited between September 2024 and March 2025 at the Complejo Asistencial Universitario de Soria (Soria, Spain). Individuals who expressed interest received a telephone call explaining the study purpose, procedures, potential benefits, and possible risks. Eligible participants provided written informed consent prior to baseline assessment.

Participants were eligible if they met the following criteria:

(i) Diagnosis of MS according to the most recent revisions of the McDonald criteria [37], regardless of phenotype (relapsing–remitting, primary progressive, or secondary progressive);
(ii) Age ≥ 18 years;
(iii) EDSS score ≤ 6 [38];
(iv) No MS exacerbation within the previous 30 days;
(v) Ability to maintain continuous walking for at least 10 min.

Participants were excluded if they:

(i) Had cognitive impairment preventing completion of the TWT protocol; Cognitive impairment was determined based on clinical judgment by the treating neurologist and review of the patient’s medical records; no formal neuropsychological screening instrument was administered specifically for study inclusion;
(ii) Were classified as at moderate or high risk for physical activity participation, operationalised as ≥3 affirmative responses on the Physical Activity Readiness Questionnaire for Everyone (PAR-Q+) [39], an internationally used pre-participation screening tool [40];
(iii) Had a history of frequent falls (≥4 falls) in the previous 6 months;
(iv) Reported severe symptoms during TWT that compromised safe participation (e.g., vertigo or dizziness).

**Figure 2:**
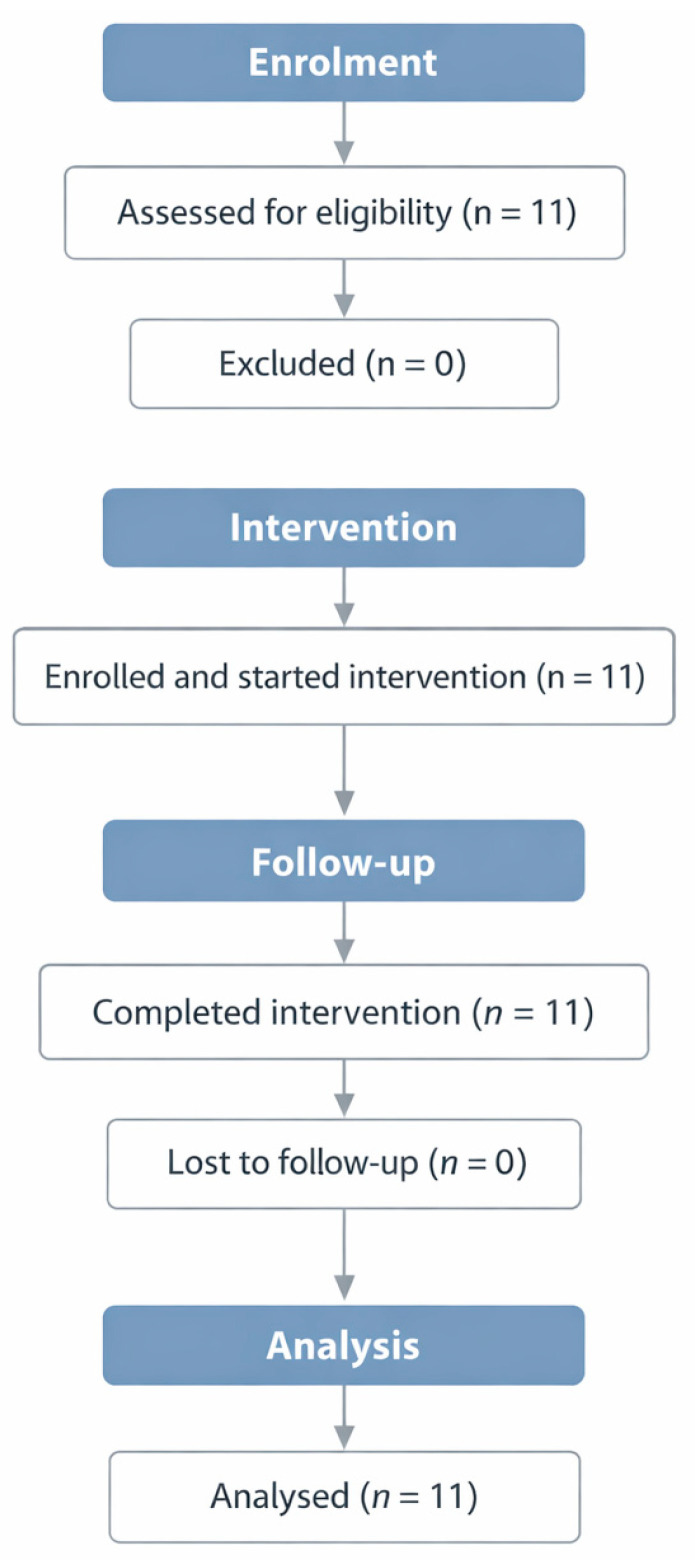
Participant flow through enrolment, intervention, and analysis in this single-arm pilot study.

All enrolled participants (*n* = 11) completed the intervention and post-intervention assessments. All participants met the predefined adherence criterion (≥80% of supervised sessions), and no participants were lost to follow-up (Figure 2).

### 2.4. Intervention

**Figure 3:**
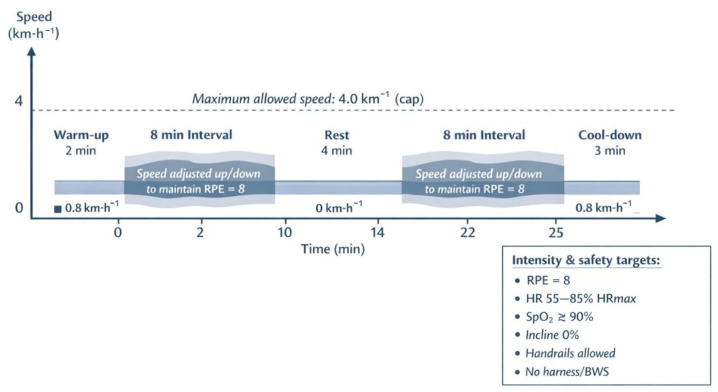
Schematic illustration of a 25 min treadmill walking training (TWT) session. The session consisted of a 2 min warm-up, two 8 min walking intervals separated by a 4 min rest, and a 3 min cool-down. During the walking intervals, treadmill speed was adjusted to maintain a target RPE of 8, within an operational range up to a maximum speed of 4.0 km·h^−1^. Safety and intensity targets are shown. Abbreviations: Km, kilometre; h, hour; min, minute; RPE, rating of perceived exertion; HR, heart rate; Hrmax, maximum heart rate; SpO_2_, peripheral oxygen saturation; BWS, body weight support.

Participants completed a 12-week TWT programme consisting of two supervised sessions per week. TWT was performed on an electric treadmill (Domyos Intense Run^®^, Decathlon, Spain; ref. 8389495). Each session lasted 25 min and comprised three phases:

1. A 2 min warm-up at 0.8 km·h^−1^;
2. A 20 min main phase consisting of two 8 min walking intervals separated by a 4 min rest period (treadmill stopped);
3. A 3 min cool-down at 0.8 km·h^−1^ (Figure 3).

During the main phase, treadmill speed was individually adjusted within an operational range (0.3–4.0 km·h^−1^) to maintain a target rating of perceived exertion (RPE) of 8, assessed verbally every minute using the Borg Category-Ratio 1–10 scale, where 1 corresponds to very light exertion and 10 to maximal exertion [41]. RPE is a recognised indicator for monitoring exercise intensity [42] and supports individualised exercise prescription [43]. This protocol was adapted from previous work in people with MS [28]. Treadmill incline was maintained at 0% throughout all sessions. Participants were allowed to use the treadmill handrails to enhance stability and reduce fall risk; no harness or body-weight support system was used.

All sessions were supervised by a qualified physiotherapist from the research team. Heart rate (HR) was monitored continuously using a chest-strap HR sensor (Garmin HRM-Pro Plus). Maximum HR (Hrmax) was estimated using the Tanaka equation (Hrmax = 208 − 0.7 × age) [44]. HR was targeted between 55% and 85% of HRmax, in accordance with American College of Sports Medicine (ACSM) recommendations [45]. Peripheral oxygen saturation (SpO_2_) was monitored using a finger pulse oximeter (OMRON, Spain). As safety criteria, exercise was temporarily paused if SpO_2_ fell below 90% or if HR exceeded 85% of HRmax; no training session required interruption based on these criteria.

Participants were instructed to maintain their usual daily routines and not to initiate additional physiotherapy, rehabilitation, or structured exercise programmes during the intervention period. No changes in medication were reported during the 12-week intervention. The progression and adjustment of treadmill speed followed a standardised RPE-guided protocol applied consistently across sessions, with individualised modifications made within sessions to maintain the target intensity.

The training frequency and duration were selected to balance feasibility, safety, and training stimulus in a clinical hospital setting. A twice-weekly schedule over 12 weeks has been commonly used in exercise interventions for people with multiple sclerosis and has been shown to be feasible and well tolerated [22,26,28]. The use of an RPE-guided intensity target was chosen to allow individualisation of training load in a population with heterogeneous disability and fatigue profiles, consistent with previous MS exercise studies and clinical recommendations [41,42,43].

### 2.5. Outcome Measures

#### 2.5.1. Quality of Life

QoL was assessed using the 36-Item Short Form Health Survey (SF-36) [46,47]. The SF-36 includes 36 items grouped into 8 domains (physical functioning, role limitations due to physical problems, bodily pain, general health, vitality, social functioning, role limitations due to emotional problems, and mental health), plus a single health transition item. Each domain score ranges from 0 (worst health status) to 100 (best health status). In addition, an overall SF-36 score (“SF-36 Total”) was computed as the arithmetic mean of the 8 domain scores. The SF-36 Total score was used as an exploratory descriptive summary of overall health status across domains. Interpretation focused primarily on domain-level results, given that component summary scores were not the primary aim of this pilot study.

#### 2.5.2. Functional Mobility (Timed up and Go)

Functional mobility was assessed using the TUG test, following procedures previously applied to people with MS [48]. Participants started seated on a chair without armrests and, on a verbal command, stood up, walked 3 m, turned around, returned to the chair, and sat down as quickly and safely as possible. Three trials were performed, and the TUG outcome was calculated as the mean of the 3 attempts. Participants were allowed to use their usual assistive devices (e.g., cane or crutch) if routinely used in daily life, and the same device was used consistently across baseline and post-intervention assessments.

#### 2.5.3. Walking Speed and Frailty Risk Indicator (4 m Walk Test)

Walking speed was assessed using a 4 m walk test performed as fast as safely possible. Two floor marks separated by 4 m defined the timed section; participants started walking 1 m before the first mark and continued walking beyond the second mark to allow for acceleration and deceleration, while only the time between the two 4 m marks was recorded. Timing was performed manually using a stopwatch. Three trials were completed, and the outcome was calculated as the mean of the three attempts. Participants were allowed to use their usual assistive devices if routinely used in daily life, and the same device was used consistently across baseline and post-intervention assessments.

As a frailty risk indicator (slowness criterion), gait speed < 0.8 m·s^−1^ was considered indicative of increased frailty-related vulnerability [14].

#### 2.5.4. Physical Function (Short Physical Performance Battery)

Physical function was assessed using the Short Physical Performance Battery (SPPB), which has been applied to people with MS [49,50]. The SPPB comprises 3 components evaluating standing balance, habitual gait speed over 4 m, and lower-limb strength. To minimise fatigue, tests were administered in the following order: (i) standing balance (side-by-side, semi-tandem, and tandem positions); (ii) 4 m walk test; and (iii) the five-times chair stand test, recorded as the time required to stand up and sit down 5 times as quickly as possible. Each component was scored according to standard SPPB criteria [50], and component scores were summed to yield a total score ranging from 0 to 12, with higher scores indicating better physical function.

#### 2.5.5. Fatigue

Fatigue was assessed using the Modified Fatigue Impact Scale (MFIS), which has been validated for people with MS [51]. The MFIS comprises 21 items evaluating the perceived impact of fatigue over the previous 4 weeks and includes 3 subscales: physical (9 items), cognitive (10 items), and psychosocial (2 items). Each item is scored from 0 to 4, with higher scores indicating a greater impact of fatigue.

### 2.6. Statistical Analysis

Statistical analyses were performed using SPSS (version 29.0.2.0). Continuous variables are reported as mean ± standard deviation (SD) when pre–post change scores were approximately normally distributed and as median [interquartile range, IQR] when change scores were non-normally distributed. Normality was assessed using the Shapiro–Wilk test applied to the within-participant change scores (T1 − T2).

Within-participant changes from baseline (T1) to post-intervention (T2) were analysed using paired Student’s *t*-tests for outcomes with normally distributed change scores and Wilcoxon signed-rank tests for outcomes with non-normally distributed change scores. All tests were two-tailed. *p*-values are reported descriptively, with a nominal threshold of *p* < 0.05, and results were interpreted with emphasis on effect size estimation given the exploratory pilot design and multiple outcomes. No adjustment for multiple comparisons was applied due to the exploratory nature of this pilot study.

Accordingly, conclusions were based on convergence of direction, magnitude of effect sizes, and consistency across related outcomes, rather than isolated *p*-values.

Effect sizes were quantified as follows: for outcomes analysed using paired *t*-tests, effect sizes are reported as Hedges’ g (bias-corrected standardised mean change). For Wilcoxon signed-rank tests, effect size was calculated as r = |Z|/√N, where N is the number of paired observations with non-zero differences.

For clarity, the direction of effect sizes reflects the direction of the computed change score (T1 − T2); therefore, positive or negative values should be interpreted in conjunction with the scaling and clinical meaning of each outcome, as specified in the table footnotes.

No missing outcome data were observed.

Given the exploratory pilot nature of the study, interpretation focused on the magnitude of effects and feasibility outcomes rather than on hypothesis testing alone.

### 2.7. Safety and Adverse Events

Potential adverse events were predefined as symptoms occurring during or immediately after training sessions, including dizziness or vertigo, palpitations or tachycardia, and musculoskeletal pain. Prior to each session, participants were asked about any discomfort and were instructed to report symptoms at any time during TWT; at the end of each session, they were systematically queried regarding adverse symptoms. No training session required interruption, and no falls or clinically relevant adverse events occurred during the 12-week intervention.

## 3. Results

### 3.1. Sample Description

Eleven participants with MS completed the 12-week TWT programme and both assessment time points (T1 and T2). Participants were predominantly female (9/11; 81.8%) and had a mean age of 51.09 ± 10.02 years. Mean time since MS diagnosis was 12.18 ± 5.91 years, and disability severity was moderate on average (EDSS score: 4.59 ± 1.45). MS phenotypes included relapsing–remitting (5/11; 45.5%), secondary progressive (4/11; 36.4%), and primary progressive disease (2/11; 18.2%). Baseline sociodemographic and clinical characteristics are presented in Table 1. Detailed baseline information on MS disease-modifying therapies, concomitant medications, and relevant medical history is provided in Supplementary Table S1.

### 3.2. Outcomes After the Intervention

#### 3.2.1. Quality of Life (SF-36)

After the 12-week TWT programme, the SF-36 total score did not change significantly (T1: 57.05 ± 16.70 vs. T2: 63.47 ± 19.03; *p* = 0.160). At the domain level, statistically significant improvements were observed in general health (T1: 38.64 ± 14.74 vs. T2: 47.73 ± 12.27; *p* = 0.039; g = −0.545) and vitality (T1: 37.50 ± 22.54 vs. T2: 53.41 ± 23.78; *p* = 0.043; g = −0.530). No significant changes were found in physical functioning, role physical, bodily pain, mental health, or the health transition item (all *p* > 0.05). For the non-normally distributed domains, role emotional and social functioning showed no significant changes (both *p* > 0.05; r = 0.255 and r = 0.450, respectively) (Table 2).

#### 3.2.2. Mobility, Gait Speed, and Physical Function

Functional mobility improved, with TUG time decreasing from 9.27 ± 3.03 s at baseline to 8.28 ± 2.84 s post-intervention (*p* = 0.007; g = 0.829). Walking performance improved, with 4 m walk test increasing from 0.79 ± 0.17 m·s^−1^ to 1.08 ± 0.30 m·s^−1^ (*p* < 0.001; g = −1.311). Physical function assessed by SPPB increased from a median [IQR] of 9 [3] at T1 to 12 [2] at T2 (*p* = 0.003; r = 0.892) (Table 3).

#### 3.2.3. Fatigue

Fatigue impact improved following the intervention. MFIS total score decreased from 45.73 ± 8.79 at T1 to 28.82 ± 15.31 at T2 (*p* = 0.015; Hedges’ g = 0.699). Significant improvements were also observed in the MFIS physical subscale (25.00 ± 4.45 to 15.18 ± 9.12; *p* = 0.007; g = 0.836) and psychosocial subscale (4.91 ± 2.07 to 2.73 ± 2.61; *p* = 0.026; g = 0.614). The cognitive subscale decreased from 15.82 ± 6.13 to 11.91 ± 9.26, but this change was not statistically significant (*p* = 0.094; g = 0.393) (Table 4).

### 3.3. Adherence to the Treadmill Walking Training Programme

Participants completed 22 of the 24 scheduled sessions, corresponding to an adherence rate of 91.7%.

### 3.4. Adverse Events

No adverse events were reported during the intervention. No sessions required interruption due to SpO_2_ falling below 90% or HR exceeding 85% of predicted HRmax.

## 4. Discussion

### 4.1. Principal Findings and Clinical Relevance

This single-arm pilot study evaluated the feasibility, safety, and observed pre–post changes associated with a 12-week TWT programme in people with MS. Given the uncontrolled exploratory design and the small sample size, the findings should be interpreted as preliminary associations rather than evidence of intervention effectiveness. The main findings were that the intervention was feasible in a hospital setting, with high adherence and complete outcome data, and no adverse events were reported. Clinically relevant outcomes—including mobility (TUG), 4 m gait speed (used here as a frailty-related slowness indicator), physical function (SPPB), and fatigue (MFIS)—showed favourable pre–post changes in this sample. The magnitude of changes observed in TUG and gait speed falls within ranges considered clinically meaningful in people with multiple sclerosis, supporting their potential functional relevance. For example, TUG performance improved by approximately 11%, gait speed increased by ~37%, and MFIS total scores decreased by ~37% from baseline to post-intervention. Given the heterogeneity and multidimensional nature of disability manifestations in multiple sclerosis, and the exploratory design of this pilot study, the interpretation of these findings benefits from a multidimensional outcome perspective. Accordingly, the present study deliberately incorporated a broad set of outcomes capturing complementary domains, including mobility (Timed Up and Go), walking performance (gait speed), physical function (Short Physical Performance Battery), fatigue (Modified Fatigue Impact Scale), and health-related quality of life (SF-36). This multidimensional assessment approach aligns with current conceptual frameworks in MS rehabilitation, which emphasise that functional limitations, symptom burden, and patient-reported outcomes represent distinct yet interrelated dimensions of disease impact rather than interchangeable constructs.

Assessing multiple domains in parallel allows a more comprehensive characterisation of intervention-associated changes and facilitates the identification of domain-specific response patterns that may be overlooked when relying on a single outcome. This approach is particularly relevant in MS, where changes in physical performance do not necessarily translate into proportional changes in fatigue perception or quality of life, and vice versa.

With respect to clinical meaningfulness, established minimal clinically important difference (MCID) thresholds are not consistently defined for all outcomes in people with multiple sclerosis, particularly in heterogeneous samples and across different disability levels [8,22]. However, available evidence suggests that reductions of approximately 10–15% in Timed Up and Go (TUG) performance and increases of around 0.1 m·s^−1^ in gait speed are often considered clinically relevant in neurological populations, including MS [48]. In this context, the magnitude of the pre–post changes observed in TUG and gait speed in the present study exceeds these commonly cited thresholds, supporting their potential functional relevance. For other outcomes, such as MFIS, SPPB, and SF-36 domains, MCIDs remain less clearly established in MS, and reported thresholds vary across studies and populations [22,31,32]. Accordingly, changes in these outcomes should be interpreted cautiously, in conjunction with effect sizes and the exploratory nature of this pilot study. Although moderate-to-large effect sizes were observed for several outcomes, these estimates warrant cautious interpretation given the small sample size and the inherent imprecision of effect size estimation in pilot studies.

In contrast, overall health-related QoL (SF-36 total score) did not change significantly, although improvements were observed in specific domains (general health and vitality). Heterogeneity in MS phenotype and disability level may have contributed to variability in individual responses to the intervention and may partly explain the domain-specific nature of the observed changes. These results support the practicality of implementing a supervised TWT protocol within routine care pathways and justify adequately powered controlled trials to confirm efficacy and estimate comparative effectiveness [4,11,52]. Importantly, the single-arm pre–post design without a control group limits causal inference; therefore, the observed pre–post changes should be interpreted as exploratory associations rather than evidence of intervention efficacy. Accordingly, effect sizes were emphasised to estimate the magnitude of observed changes and to inform the design of future trials.

### 4.2. Quality of Life: Domain-Specific Changes and Measurement Considerations

Although the SF-36 total score did not change significantly, significant pre–post changes in general health and vitality were observed, which may reflect changes in perceived health status and energy levels—domains that are plausibly responsive to structured exercise in MS [4,11]. It should be noted that the *p*-values observed for general health and vitality were marginal and should be interpreted cautiously in the context of multiple comparisons and the exploratory nature of this pilot study. As no adjustment for multiple testing was applied, these findings should be viewed as hypothesis-generating rather than confirmatory and considered alongside effect sizes and consistency with prior literature rather than statistical significance alone, in line with recommendations for exploratory pilot studies [34,35]. The absence of a significant change in overall QoL may reflect several factors: (i) limited statistical power in a small pilot sample; (ii) heterogeneity in MS phenotype and disability, which can dilute patient-reported outcomes; and (iii) the choice of a generic QoL instrument. Although the SF-36 is widely validated and facilitates comparisons across conditions, it may be less sensitive to disease-specific changes in MS. Disease-specific QoL measures (e.g., MSQoL instruments) may be more sensitive to change in MS, as shown by variability across prior trials using MS-specific scales [8,22]. Moreover, ceiling effects in certain SF-36 domains (e.g., role emotional) may limit responsiveness, particularly in small samples. Taken together, our findings suggest that QoL effects may be domain-specific and dependent on measurement strategy, intervention dose, and participant phenotype [8,22,31]. Accordingly, interpretation was centred on domain-level changes rather than the SF-36 Total score.

The SF-36 was selected because it is a widely validated instrument, available in Spanish, and allows comparison across clinical populations [46,47]. However, MS-specific quality-of-life instruments, such as the MSQOL-54, may be more sensitive to disease-related changes in people with multiple sclerosis and should be considered in future trials [31,32].

### 4.3. Fatigue: Potential Mechanisms and Comparison with the Literature

Fatigue is among the most disabling MS symptoms and is strongly linked to reduced activity and participation [11,12,13,17,53]. In this study, MFIS total and selected subscales decreased after TWT, while the cognitive subscale did not reach statistical significance. These mixed subscale results are plausible, as fatigue is multifactorial and may respond differently across physical and cognitive dimensions [12]. Our findings align with prior work reporting fatigue improvements following exercise-based interventions in MS, including treadmill-based protocols of similar duration [22], while shorter interventions may provide insufficient stimulus to elicit measurable change [8]. Differences across studies may also reflect baseline disability, outpatient versus inpatient status, and the fatigue instrument used [8,22,26]. Importantly, perceived exertion is often disproportionately high in MS relative to physiological workload [26,54], making RPE-guided prescription clinically appealing. In this context, maintaining a target RPE may help to standardise perceived intensity across individuals with varying disability and fatigue profiles, potentially supporting adherence and tolerability [41,42,43].

### 4.4. Walking Performance and Functional Mobility: Implications for Falls-Related Outcomes

Walking impairment is a major contributor to disability and reduced independence in MS [10,55,56]. The observed improvements in TUG and 4 m gait speed are consistent with the concept of task-specific training and repetitive practice enhancing motor control and functional capacity [24,29].

The observed pre–post changes in TUG and 4 m gait speed are consistent with the concept of task-specific training and repetitive practice enhancing motor control and functional capacity [24,29]. Improvements in TUG are particularly relevant because slower performance has been associated with fall risk and mobility limitations in MS [48,57,58]. The magnitude of the increase in gait speed observed in this study appears larger than that reported in some prior treadmill-based interventions in people with MS, where more modest gains have typically been described [8,9,22,26,28]. However, differences across studies may be explained by variability in baseline disability, training dose and duration, supervision intensity, and outcome assessment protocols [8,9,22]. In addition, the relatively low baseline gait speed of the present sample may have allowed greater room for improvement, a phenomenon commonly observed in rehabilitation studies involving participants with reduced initial performance. Nevertheless, given the single-arm pre–post design, the potential contribution of test–retest learning effects or familiarisation with the walking assessments cannot be excluded and may partly account for the observed changes [34]. This consideration further underscores the need for controlled designs with appropriate comparison groups to disentangle true training-related adaptations from nonspecific or practice-related effects [34]. Given the high prevalence of falls and fear of falling in MS [16,52,57], even modest gains in functional mobility may have meaningful downstream implications for confidence and participation. While some prior treadmill-based interventions did not report significant changes in TUG [9], discrepancies may be explained by differences in training dose and duration, participant disability, and programme components [9]. Mechanistically, repeated gait cycle practice may facilitate adaptive changes in neuromuscular coordination and compensatory neuroplasticity within remaining neural networks, as hypothesised for task-specific rehabilitation approaches [24,59].

### 4.5. Frailty-Related Risk Indicator and Physical Function (SPPB): Positioning the Contribution

Frailty is increasingly recognised as clinically relevant in MS, where reduced reserves may be driven by chronic inflammation, neurodegeneration, and reduced physical activity [7,60]. In this pilot study, we used 4 m gait speed as a frailty-related slowness indicator, applying a commonly used threshold (<0.8 m·s^−1^) [14]. The observed increase in gait speed suggests a favourable shift in this risk indicator; however, it should be emphasised that frailty was not assessed as a multidimensional construct. This distinction is important for interpretation and for framing future studies that incorporate validated frailty indices alongside mobility outcomes [7,14].

Physical function, assessed with the SPPB, showed favourable pre–post changes after TWT. The SPPB integrates balance, gait speed, and chair-stand performance and has been used in MS populations [49,50]. Balance impairments are common in MS and contribute to fall risk [15]. Evidence indicates that interventions incorporating functional training, balance, and gait-related practice may yield the most consistent balance improvements [61], and TWT may contribute to this effect via repeated stepping practice and reduced fear during supervised training.

### 4.6. Feasibility, Safety, and Implementation in Routine Care

A key strength of this work is the demonstration of feasibility within a real hospital context, supported by high adherence, no attrition, complete outcome data, and the absence of adverse events. These features are particularly relevant for implementation within public health services, where scalability and safety monitoring are essential. Supervision by qualified staff and predefined safety thresholds for HR and SpO_2_ likely contributed to tolerability and risk mitigation [45]. From a service perspective, a structured, time-limited protocol with clear intensity targets (RPE-guided adjustments) may facilitate standardisation across clinicians and patient subgroups [41,42,43]. The absence of dropouts may reflect the supervised hospital-based setting, close monitoring, and the inclusion of participants able to walk continuously for at least 10 min; however, selection bias cannot be excluded and may limit generalisability [16,22,45].

### 4.7. Limitations, Strengths, and Future Research

This study has limitations inherent to its pilot design. First, the single-arm pre–post design without a control or comparison group limits causal inference; observed changes may partly reflect nonspecific effects of attention, regression to the mean, or test–retest learning effects, particularly in mobility assessments. Second, the sample size was small and heterogeneous with respect to MS phenotype and disability severity, limiting statistical precision, reducing generalisability, and increasing the risk of type I error across multiple outcomes. Multiple outcomes were analysed without adjustment for multiple comparisons. Although this approach is acceptable in exploratory pilot research, it increases the risk of type I error; therefore, statistically significant findings should be interpreted cautiously and in conjunction with effect sizes and clinical relevance. Additionally, the sample consisted predominantly of women, which may limit generalisability and precludes exploration of potential sex-related differences in response to treadmill walking training. The relatively wide age range of participants may also have contributed to variability in individual training responses and should be considered when interpreting the findings. Third, assessor blinding was not feasible in this open-label pilot study, which may have introduced expectation bias in performance-based measures. Fourth, although clinically feasible, the reliance on manual timing for gait and mobility tests may introduce measurement error compared with instrumented assessment systems (e.g., inertial measurement unit–based sensors during the Timed Up and Go test). Fifth, quality of life was assessed using the generic SF-36 instrument, which may be less sensitive to MS-specific changes than disease-targeted tools [8,22,31,32]. Sixth, frailty was not assessed as a multidimensional clinical syndrome but was operationalised solely through gait speed (<0.8 m/s as a slowness indicator), which does not capture the complexity of frailty [7,14]. Finally, the absence of follow-up assessments precludes evaluation of the durability of observed changes beyond the immediate post-intervention period, which is particularly relevant given that training effects may attenuate once the programme ends [30].

Despite these limitations, the study has several strengths. It was conducted in a real-world hospital setting, demonstrating practical feasibility and safety with high adherence, no attrition, and complete data capture—features that support implementation potential. The intervention was clearly structured, supervised, and delivered with prespecified safety monitoring, enhancing reproducibility. The outcome battery captured complementary dimensions (mobility, walking performance, physical function, fatigue, and QoL) using widely used instruments in MS research and clinical practice [46,47,48,49,50,51,62,63].

Future trials should prioritise controlled designs to establish efficacy and inform clinical decision-making. Specifically, adequately powered randomised controlled trials should compare treadmill walking training with usual care and/or active control interventions (e.g., overground walking or multicomponent exercise), with stratification by MS phenotype and disability level (EDSS) to evaluate potential effect modification. Follow-up assessments are needed to determine the maintenance of observed benefits and to evaluate strategies that support long-term adherence (e.g., transition to home-based programmes). Outcomes should include MS-specific quality-of-life instruments, objective physical activity monitoring (free-living walking), and falls-related endpoints (falls incidence, fear of falling) to better capture functional impact [16,52,57]. Where feasible, objective gait assessment using instrumented measures and blinded outcome assessment should be incorporated to reduce measurement bias. Finally, dose–response analyses (frequency, duration, intensity targets) and combinations with adjunct therapies such as virtual reality may help optimise programme effectiveness and scalability [8,9].

### 4.8. Practical Applications

In routine neurorehabilitation settings, a supervised TWT programme can be delivered safely to ambulatory adults with MS (EDSS ≤ 6) who can walk continuously for ≥10 min, using a standardised dose of two 25 min sessions per week over 12 weeks. Individualising treadmill speed through frequent RPE (target RPE ≈ 8) while monitoring HR (55–85% of predicted HRmax) and SpO_2_ offers a pragmatic framework to balance training stimulus and safety, without requiring harness systems and permitting handrail use as needed. Clinicians can track response using brief, clinically feasible measures such as the TUG, 4 m gait speed, SPPB, and MFIS to guide progression and identify patients who may benefit most. It should be emphasised that this protocol was implemented in a supervised hospital setting with continuous safety monitoring; its direct translation to unsupervised or home-based environments requires further evaluation of safety, efficacy, and adherence support strategies.

## 5. Conclusions

In this pilot study, the observed pre–post changes should be interpreted cautiously and considered preliminary, pending confirmation in adequately powered randomised controlled trials. Walking impairment and fatigue are highly prevalent in MS and contribute to reduced physical function and QoL. In this single-arm pilot study, a 12-week supervised TWT programme was feasible, safe, and well tolerated, with high adherence and no adverse events. Favourable pre–post changes were observed in functional mobility (TUG), gait speed (used here as a frailty-related slowness indicator), physical function (SPPB), and fatigue (MFIS). In contrast, the SF-36 total score did not change significantly, although significant pre–post changes were noted in the general health and vitality domains. These preliminary findings underscore the feasibility of implementing supervised TWT in routine neurorehabilitation and justify further evaluation through adequately powered randomised controlled trials with longer follow-up, multidimensional frailty assessment, and patient-centred endpoints to clarify its efficacy, durability, and clinical impact.

## Figures and Tables

**Table 1:** Baseline sociodemographic and clinical characteristics of participants (*n* = 11).

Characteristic	Value
Age, years	51.09 ± 10.02
Sex, *n* (%)	
Female	9 (81.8)
Male	2 (18.2)
Years since MS diagnosis	12.18 ± 5.91
Weight, kg	67.18 ± 10.42
Height, m	1.61 ± 0.09
Certified disability (official), %	32.0 ± 26.78
EDSS score	4.59 ± 1.45
MS phenotype, *n* (%)	
Relapsing–remitting	5 (45.5)
Secondary progressive	4 (36.4)
Primary progressive	2 (18.2)
Disease-modifying therapy, *n* (%)	
On DMT	8 (72.7)
No DMT	3 (27.3)
Education level, *n* (%)	
Primary or lower	2 (18.2)
Secondary/vocational	4 (36.4)
University	5 (45.5)
Employment status, *n* (%)	
Employed	5 (45.5)
Unemployed	3 (27.3)
Work disability	3 (27.3)
Smoking status, *n* (%)	
Never	6 (54.5)
Current	3 (27.3)
Former	2 (18.2)
Alcohol consumption, *n* (%)	
No	11 (100.0)
Sleep-related variables, *n* (%)	
Difficulty initiating sleep (yes)	1 (9.1)
Sleep medication (yes)	2 (18.2)
Self-rated sleep quality, *n* (%)	
Difficulty initiating sleep	1 (9.1)
Sleep medication use	2 (18.2)
Self-rated sleep quality (normal–very satisfactory)	9 (81.8)

Data are presented as mean ± SD or *n* (%). Abbreviations: EDSS, Expanded Disability Status Scale; MS, multiple sclerosis; SD, standard deviation; DMT, disease-modifying therapy.

**Table 2:** Pre–post intervention assessment of quality of life (SF-36).

SF-36 Domain	T1	T2	Mean Difference (T1 − T2)	Test	Effect Size	*p*-Value
SF-36 Total	57.05 ± 16.70	63.47 ± 19.03	−6.42 ± 20.37	t = −1.046	−0.291	0.160
Physical functioning	52.73 ± 23.70	61.82 ± 27.23	−9.09 ± 20.95	t = −1.439	−0.400	0.090
Role physical	52.84 ± 30.15	61.93 ± 25.23	−9.09 ± 36.48	t = −0.826	−0.230	0.214
Bodily pain	47.50 ± 29.00	56.14 ± 37.59	−8.64 ± 29.44	t = −0.973	−0.271	0.177
General health	38.64 ± 14.74	47.73 ± 12.27	−9.09 ± 15.40	t = −1.958	−0.545	0.039 *
Vitality	37.50 ± 22.54	53.41 ± 23.78	−15.91 ± 27.72	t = −1.903	−0.530	0.043 *
Mental health	70.00 ± 16.58	67.27 ± 26.96	2.73 ± 28.14	t = 0.321	0.089	0.377
Health transition (current health)	38.64 ± 34.21	45.45 ± 29.19	−6.82 ± 19.66	t = −1.150	−0.320	0.138
Role emotional †	100.00 [33.33]	100.00 [41.67]	—	Z = −0.845	r = 0.255	0.398
Social functioning †	75.00 [50.00]	87.50 [50.00]	—	Z = −1.492	r = 0.450	0.136

Data are presented as mean ± SD unless otherwise indicated. † Data are presented as median [IQR] and analysed using the Wilcoxon signed-rank test; mean differences are not reported for these outcomes. Mean difference is calculated as T1 − T2. For SF-36 domains, higher scores indicate better health status; therefore, negative mean differences indicate favourable pre–post changes. Effect sizes are reported as Hedges’ g for paired *t*-tests and as r = |Z|/√*n* for Wilcoxon signed-rank tests (*n* = number of paired observations with non-zero differences). * *p* < 0.05.

**Table 3:** Pre–post intervention assessment of mobility, walking performance, and physical function.

Outcome	T1	T2	Mean Difference (T1 − T2)	Test	Effect Size	*p*-Value
TUG, s	9.27 ± 3.03	8.28 ± 2.84	0.99 ± 1.10	t = 2.980	0.829	0.007 *
4 m gait speed, m·s^−1^	0.79 ± 0.17	1.08 ± 0.30	−0.29 ± 0.20	t = −4.714	−1.311	<0.001 *
SPPB total score †	9 [3]	12 [2]	—	Z = −2.958	r = 0.892	0.003 *

Abbreviations: TUG: Timed Up and Go; SPPB: Short Physical Performance Batter. Data are presented as mean ± SD unless otherwise indicated. Data are presented as median [IQR] and were analysed using the Wilcoxon signed-rank test; mean differences are not reported for these outcomes. Mean difference is calculated as T1 − T2. For TUG, lower values indicate better performance; therefore, positive mean differences indicate favourable pre–post changes. For 4 m gait speed, higher values indicate better performance; therefore, negative mean differences indicate favourable pre–post changes. Effect sizes are reported as Hedges’ g for *t*-tests and r = |Z|/√*n* for Wilcoxon signed-rank tests. * *p* < 0.05. † Data are presented as median [IQR] and analysed using the Wilcoxon signed-rank test.

**Table 4:** Pre–post intervention assessment of fatigue (MFIS).

Outcome	T1	T2	Mean Difference (T1 − T2)	Test	Effect Size	*p*-Value
MFIS Total	45.73 ± 8.79	28.82 ± 15.31	15.91 ± 21.02	t = 2.511	0.699	0.015 *
MFIS Physical	25.00 ± 4.45	15.18 ± 9.12	9.82 ± 10.84	t = 3.003	0.836	0.007 *
MFIS Cognitive	15.82 ± 6.13	11.91 ± 9.26	3.91 ± 9.17	t = 1.414	0.393	0.094
MFIS Psychosocial	4.91 ± 2.07	2.73 ± 2.61	2.18 ± 3.28	t = 2.206	0.614	0.026 *

Abbreviations: MFIS: Modified Fatigue Impact Scale. Data are presented as mean ± SD. Mean difference is calculated as T1 − T2; positive values indicate favourable pre–post changes (lower MFIS scores indicate less fatigue). Paired Student’s t-test was used. Effect sizes are reported as Hedges’ g. * *p* < 0.05.

## Data Availability

Not Available.

## Supplementary Materials

The following supporting information can be downloaded at https://www.mdpi.com/article/10.3390/healthcare14040552/s1, Table S1: Baseline disease-modifying therapy and concomitant clinical characteristics (*n* = 11).

## Abbreviations

The following abbreviations are used in this manuscript:

ACSM	American College of Sports Medicine
CEIC	Clinical Ethics and Research Committee
CONSORT	Consolidated Standards of Reporting Trials
EDSS	Expanded Disability Status Scale
HR	Heart rate
HRmax	Maximum heart rate
IQR	Interquartile range
MFIS	Modified Fatigue Impact Scale
MS	Multiple sclerosis
PAR-Q+	Physical Activity Readiness Questionnaire for Everyone
QoL	Quality of life
RPE	Rating of Perceived Exertion
SD	Standard deviation
SF-36	36-Item Short Form Health Survey
SpO_2_	Peripheral oxygen saturation
SPPB	Short Physical Performance Battery
TUG	Timed Up and Go
TWT	Treadmill walking training
WHO	World Health Organization

## Appendix A

The Ethics Committee of the University of Leon (Spain) Approved the Trial Protocol as Indicated by the Approval Code ETICA-ULE-010-2020.

## Appendix B

Questionnaire completed before intervention.

## Appendix C

Consolidated Standards of Reporting Trials (CONSORT) 2010 for parallel group randomised trials 2010 checklist [35].

**Section/Topic**	**Item No**	**Checklist Item**	**Reported on Page Nº**
Title and abstract			
	1a	Identification as a randomised trial in the title	-
	1b	Structured summary of trial design, methods, results, and conclusions (for specific guidance see CONSORT for abstracts)	1–2
Introduction			
Background and objectives	2a	Scientific background and explanation of rationale	2–3
	2b	Specific objectives or hypotheses	3
Methods			
Trial design	3a	Description of trial design (such as parallel, factorial) including allocation ratio	3–4
	3b	Important changes to methods after trial commencement (such as eligibility criteria), with reasons	-
Participants	4a	Eligibility criteria for participants	4–5
	4b	Settings and locations where the data were collected	3
Interventions	5	The interventions for each group with sufficient details to allow replication, including how and when they were actually administered	5–6
Outcomes	6a	Completely defined pre-specified primary and secondary outcome measures, including how and when they were assessed	6–7
	6b	Any changes to trial outcomes after the trial commenced, with reasons	-
Sample size	7a	How sample size was determined	4
	7b	When applicable, explanation of any interim analyses and stopping guidelines	-
Randomisation:			
Sequence generation	8a	Method used to generate the random allocation sequence	-
	8b	Type of randomisation; details of any restriction (such as blocking and block size)	-
Allocation concealment mechanism	9	Mechanism used to implement the random allocation sequence (such as sequentially numbered containers), describing any steps taken to conceal the sequence until interventions were assigned	-
Implementation	10	Who generated the random allocation sequence, who enrolled participants, and who assigned participants to interventions	-
Blinding	11a	If done, who was blinded after assignment to interventions (for example, participants, care providers, those assessing outcomes) and how	-
	11b	If relevant, description of the similarity of interventions	-
Statistical methods	12a	Statistical methods used to compare groups for primary and secondary outcomes	-
	12b	Methods for additional analyses, such as subgroup analyses and adjusted analyses	7–8
Results			
Participant flow (a diagram is strongly recommended)	13a	For each group, the numbers of participants who were randomly assigned, received intended treatment, and were analysed for the primary outcome	5
	13b	For each group, losses and exclusions after randomisation, together with reasons	5
Recruitment	14a	Dates defining the periods of recruitment and follow-up	4
	14b	Why the trial ended or was stopped	-
Baseline data	15	A table showing baseline demographic and clinical characteristics for each group	8–9
Numbers analysed	16	For each group, number of participants (denominator) included in each analysis and whether the analysis was by original assigned groups	5
Outcomes and estimation	17a	For each primary and secondary outcome, results for each group, and the estimated effect size and its precision (such as 95% confidence interval)	9–11
	17b	For binary outcomes, presentation of both absolute and relative effect sizes is recommended	-
Ancillary analyses	18	Results of any other analyses performed, including subgroup analyses and adjusted analyses, distinguishing pre-specified from exploratory	9–11
Harms	19	All important harms or unintended effects in each group (for specific guidance see CONSORT for harms)	11
Discussion			
Limitations	20	Trial limitations, addressing sources of potential bias, imprecision, and, if relevant, multiplicity of analyses	13
Generalisability	21	Generalisability (external validity, applicability) of the trial findings	11–13
Interpretation	22	Interpretation consistent with results, balancing benefits and harms, and considering other relevant evidence	11–13
Other information			
Registration	23	Registration number and name of trial registry	3
Protocol	24	Where the full trial protocol can be accessed, if available	3
Funding	25	Sources of funding and other support (such as supply of drugs), role of funders	15
