# Supplementary material for: Feasibility and Pre–Post Changes Associated with a 12-Week Treadmill Walking Training Programme on Walking Performance, Physical Function, Fatigue, and Quality of Life in People with Multiple Sclerosis: A Single-Arm Pilot Study
Source: Healthcare (Basel). 2026 Feb 23;14(4):552. doi: 10.3390/healthcare14040552 (PMC12940560; doi:10.3390/healthcare14040552)
Supplement: Supplementary file 1 [file healthcare-14-00552-s001.zip › healthcare-4158977-supplementary.pdf]

Table S1. Baseline disease-modifying therapy and concomitant clinical characteristics (*n* = 11)

Disease-modifying therapy (DMT) for multiple sclerosis	n (%)
No disease-modifying therapy	3 (27.3)
Ocrelizumab (Ocrevus®)	4 (36.4)
Siponimod (Mayzent®)	1 (9.1)
Dimethyl fumarate (Tecfidera®)	1 (9.1)
Cladribine (Mavenclad®)	1 (9.1)
Teriflunomide (Aubagio®)	1 (9.1)
Concomitant medications (non-MS)	
No concomitant medication reported	3 (27.3)
Gastrointestinal + nervous system + analgesics	1 (9.1)
Urological + cardiovascular + nervous system	3 (27.3)
Nervous system medications	3 (27.3)
Gastrointestinal + metabolic + urological + nervous system	1 (9.1)
Relevant medical history	
No relevant medical history reported	2 (18.2)
Neurological disorders (other than MS)	2 (18.2)
Previous surgical procedures	3 (27.3)
Cardiovascular + urological disorders	1 (9.1)
Traumatological disorders	1 (9.1)
Cardiovascular + neurological disorders	2 (18.2)

Data are presented as n (%).

Abbreviations: DMT, disease-modifying therapy; MS, multiple sclerosis.

Categories reflect self-reported medical history and medication use recorded at baseline using the structured questionnaire.
